# Etiology of Pervasive Versus Situational Antisocial Behaviors: A Multi‐Informant Longitudinal Cohort Study

**DOI:** 10.1111/cdev.12456

**Published:** 2015-11-12

**Authors:** Jasmin Wertz, Helena M. S. Zavos, Timothy Matthews, Rebecca Gray, Janis Best‐Lane, Carmine M. Pariante, Terrie E. Moffitt, Louise Arseneault

**Affiliations:** ^1^ King's College London; ^2^ Duke University

## Abstract

The aim of this study was to disentangle pervasive from situational antisocial behaviors using multiple informants, and to investigate their genetic and environmental etiologies in preadolescence and across time. Antisocial behaviors were assessed in 2,232 twins from the Environmental Risk (E‐Risk) Longitudinal Twin Study at ages 5 and 12. Pervasive antisocial behaviors were defined as behaviors that mothers, teachers, interviewers, and twins themselves agreed on. Results from a psychometric model indicated that the variation in children's pervasive antisocial behaviors was mostly accounted for by familial influences that originated in childhood, whereas situational behaviors were explained by newly emerging nonshared environmental and genetic influences. This study shows that children's pervasive and situational antisocial behaviors have distinct etiologies that could guide research and treatment.

Children who show antisocial behaviors in some situations may not behave antisocially in others (De Los Reyes, Henry, Tolan, & Wakschlag, 2009; Dirks, De Los Reyes, Briggs‐Gowan, Cella, & Wakschlag, 2012). For example, a child may fight at home with siblings or argue with parents but be cooperative and well behaved at school. Few children display antisocial behaviors that are pervasive, that is, shown across different settings (De Los Reyes et al., 2009). Such pervasive behaviors signal more severe problems (American Psychiatric Association [APA], 2013; Frick & Nigg, 2012) and are thought to be associated with a trajectory of lifelong persistent criminality (Moffitt, 1993). Distinguishing pervasive from situational antisocial behaviors has clinical utility for determining treatment need as it helps estimate the severity of antisocial behavior problems and predict later prognosis. However, these behavior patterns are rarely considered separately for testing etiological hypotheses about antisocial behaviors. The present study aims to fill this gap by examining the origins of pervasive and situational antisocial behaviors in preadolescence, using a multi‐informant, longitudinal, genetically sensitive design.

We know very little about the etiology of situational versus pervasive antisocial behaviors, but there are several reasons why antisocial behaviors displayed across settings could have a different etiology from behaviors that are displayed in only some situations. Children may behave antisocially in some situations because they respond to an environmental influence toward antisocial behaviors in one setting, such as a conflictual relationship with their siblings or harsh parenting (Dirks, Treat, & Weersing, 2010; Treutler & Epkins, 2003). These children may be able to curb the inclination to behave antisocially when they are in a highly structured environment, for example, in school. The etiology of situational antisocial behaviors would therefore strongly reflect characteristics of a situation or of the people a child interacts with. Although it is clear that how children react to and create situations is to some extent under genetic influence (Moffitt, 2005), we would therefore expect a larger proportion of the individual differences in situational antisocial behaviors to be accounted for by environmental influences.

In contrast, antisocial behaviors that are expressed across a variety of settings may signal that a child exercises little control over his or her behaviors and is less sensitive or responsive to the demands of a situation. The available evidence suggests that such pervasive antisocial behaviors are indicative of a significant genetic risk, with more serious antisocial behaviors concentrated in families (Farrington, Jolliffe, Loeber, Stouthamer‐Loeber, & Kalb, 2001). Consistent with these findings, antisocial behaviors that are agreed upon by informants from different settings are strongly genetically influenced in children at school entry (Arseneault et al., 2003). Our aim was to test whether and how etiological influences change in preadolescence, because there is little knowledge on the etiology of pervasive antisocial behaviors in this age group. The majority of previous studies examined antisocial behaviors in early childhood or in adolescence (Moffitt, 2005), which are the age periods that most developmental theories of crime focus on (Moffitt, 1993; Sampson & Laub, 2005; Tremblay, 2010). However, preadolescence is a dynamic developmental period, characterized by the transition from childhood to adolescence and its accompanying social, psychological, and biological maturation. It brings with it a range of new experiences and opportunities, as children begin to spend more time outside the home with their peer groups and in neighborhoods, and experience new conflicts with their parents when negotiating their independence. Changes in schools, peer dynamics, and Parent–Child interactions may translate into an increased importance of shared and nonshared environment. Therefore, although we expected genetic influences to still play a significant role in the etiology of pervasive antisocial behaviors during this time, we also predicted a contribution of shared and nonshared environment, reflecting the importance of children's social context and individual experiences in influencing antisocial behaviors at the beginning of adolescence.

Pervasive antisocial behaviors often indicate problematic behaviors that emerge in early childhood and are persistent across time (Farrington, 2005; Moffitt, Caspi, Harrington, & Milne, 2002). We therefore adopted a longitudinal approach, in order to obtain a comprehensive picture of the genetic and environmental influences underpinning pervasive antisocial behaviors across time. Examining antisocial behaviors at more than one time point makes it possible to explore the relative importance of genetic and environmental influences in explaining the stability of antisocial behaviors. It also allows for the testing of the hypothesis that genetic and environmental influences on pervasive antisocial behaviors in preadolescence originate earlier in life. In keeping with the prediction that severe and pervasive antisocial behaviors are the products of children's neuropsychological abnormalities and early environmental risk factors such as inadequate parenting and poverty (Moffitt, 1993), we expected the genetic and environmental influences on pervasive antisocial behaviors to show considerable stability. In other words, we expected the influences on pervasive behaviors at age 12 to mostly originate in early life, with a smaller role for newly emerging influences during preadolescence. For situational behaviors, we expected to find mainly transient influences, but also some stability in so far as genetic and environmental influences may reflect characteristics that influence situational antisocial behaviors across time.

Boys display higher levels of pervasive, severe, and early‐onset conduct problems (Moffitt et al., 2002). There is some evidence supporting sex differences in the etiology of antisocial behaviors (Bartels, van de Aa, van Beijsterveldt, Middeldorp, & Boomsma, 2011; Hudziak et al., 2003), although a meta‐analysis that aggregated findings across age and informants did not report any gender differences (Rhee & Waldman, 2002). It is possible that sex differences in the etiology of antisocial behaviors vary across development. Preadolescent boys and girls differ in the timing of the maturation of their bodies and brains (Koolschijn & Crone, 2013; Tanner, 1981). They also experience differential parenting during this time, with girls being monitored more by their parents compared to boys (Racz & McMahon, 2011). These differences may translate into sex differences in the magnitude of etiological influences. Furthermore, there are findings to suggest that gender differences may be most pronounced when persistent and pervasive antisocial behaviors are isolated (Fontaine, Rijsdijk, McCrory, & Viding, 2010). We therefore explored sex differences in the magnitude of genetic and environmental influences on boys' and girls' preadolescent antisocial behaviors.

The aim of this study was to investigate the genetic and environmental influences on pervasive and situational antisocial behaviors and their stability across childhood, and to examine the extent to which these influences originate earlier in life. We used a multi‐informant design to isolate pervasive antisocial behaviors. Integrating information from multiple informants to evaluate pervasiveness is recommended by the *Diagnostic and Statistical Manual of Mental Disorders* (*DSM–V*) for establishing diagnoses of attention deficit hyperactivity and conduct disorder (APA, 2013). There is abundant evidence to support this approach: It has been shown that reports from different informants complement each other, and that some of the low agreement between informants is due to them observing children in different contexts, rather than rater bias (Arseneault et al., 2003; Bartels et al., 2003; De Los Reyes et al., 2009; Hudziak et al., 2003). In addition, antisocial behaviors that different informants agree on are associated with more severe childhood maladjustment and with worse adult outcomes (Drugli, Larsson, Clifford, & Fossum, 2007; Moffitt et al., 2002).

## Method

### Sample

Participants were members of the Environmental Risk (E‐Risk) Longitudinal Twin Study, which tracks the development of a nationally representative cohort of 2,232 British children. The sample was drawn from a larger birth registry of twins born in England and Wales from 1994 through 1995 (Trouton, Spinath, & Plomin, 2002). Details about the sample have been reported previously (Moffitt & E‐Risk Study Team, 2002). Briefly, the E‐Risk sample was constructed from 1999 through 2000, when 1,116 families with same‐sex 5‐year‐old twins (93% of those eligible) participated in home‐visit assessments. Families were recruited to represent the U.K. population of families with newborns in the 1990s, based on residential location throughout England and Wales and mother's age (i.e., older mothers having twins via assisted reproduction were underselected and teenage mothers with twins were overselected). We used this sampling to replace high‐risk families who were selectively lost to the register via nonresponse and to ensure sufficient numbers of children growing up in high‐risk environments. Follow‐up home visits were conducted when the children were aged 7 (98% participation), 10 (96%), and 12 (96%) years. The sample includes 55% monozygotic (MZ) and 45% dizygotic (DZ) twin pairs. Sex is evenly distributed within zygosity (49% were boys). Parents gave informed consent and children gave assent. Ethical approval was granted by the Joint South London and Maudsley and the Institute of Psychiatry NHS Ethics Committee.

At follow‐up, the study sample represents the full range of socioeconomic conditions in the United Kingdom, as reflected in the families' distribution on a neighborhood‐level socioeconomic index ACORN [A Classification of Residential Neighborhoods], developed by CACI Inc. for commercial use in Great Britain; Odgers, Caspi, Russell, et al., 2012). ACORN uses census and other survey‐based geodemographic discriminators to classify enumeration districts (~150 households) into socioeconomic groups ranging from “wealthy achievers” (Category 1) with high incomes, large single‐family houses, and access to many amenities, to “hard‐pressed” neighborhoods (Category 5) dominated by government‐subsidized housing estates, low incomes, high unemployment, and single parents. ACORN classifications were geocoded to match the location of each E‐Risk Study family's home (Odgers, Caspi, Bates, Sampson, & Moffitt, 2012). E‐Risk families' ACORN distribution closely matches that of households nationwide: 25.6% of E‐Risk families live in “wealthy achiever” neighborhoods compared to 25.3% nationwide; 5.3% versus 11.6% live in “urban prosperity” neighborhoods; 29.6% versus 26.9% live in “comfortably off” neighborhoods; 13.4% versus 13.9% live in “moderate means” neighborhoods; and 26.1% versus 20.7% live in “hard‐pressed” neighborhoods. E‐Risk underrepresents “urban prosperity” because such households are significantly more likely to be childless.

### Antisocial Behaviors

*Mothers' reports* at ages 5 and 12 were obtained in interviews using the Child Behavior Checklist (CBCL; Achenbach, 1991a). We used the Delinquency and Aggression scales supplemented with *DSM–IV* items (APA, 1994) assessing conduct and oppositional defiant disorder (e.g., “spiteful, tries to get revenge,” “uses force to take something from another child”). The internal consistency reliabilities of the mothers' reports were 0.92 at age 5 and 0.94 at age 12. Data were available for 99.9% of the total sample at age 5 (*N* = 2,230) and 96% at age 12 (*N* = 2,141).

*Teachers' reports* at ages 5 and 12 were obtained using the Teacher Report Form (TRF; Achenbach, 1991b) supplemented as above. The behaviors of both twins were rated by the same teacher for 79% of the twins at age 5 and 30% at age 12. The internal consistency reliability of the teachers' reports was 0.95 at age 5 and 0.96 at age 12. Data were available for 94% of the total sample at age 5 (*N* = 2,091) and 79% at age 12 (*N* = 1,767).

#### Interviewers' Observations

After the home visit at age 5, interviewers rated each twin on the Dunedin Behavioural Observation Scale, which includes nine items measuring disruptive behaviors (e.g., hostility, lability, roughness; Caspi, Henry, McGee, Moffitt, & Silva, 1995). Each behavior was defined in explicit terms, and the interviewer evaluated whether each characteristic was observed *not at all* (0), *somewhat* (1), or *definitely* (2). The same interviewer rated both twins. The internal consistency reliability was 0.90 and the interrater reliability coefficient was 0.70. Data were available for 99.7% of the total sample (*N* = 2,225). When twins were aged 12, interviewers rated each twin on a child version of the Big Five Inventory (Digman & Shmelyov, 1996; Goldberg, 2001). Antisocial behaviors were operationalized using items such as rudeness, spitefulness, and anger from the agreeableness subscale of the inventory. Each item was defined in terms of a set of specific behaviors explaining the concept, and the interviewer evaluated whether each characteristic was observed *not at all* (0), *somewhat* (1), or *definitely* (2). We reverse coded the scale so that higher scores indicate more antisocial behaviors. The same interviewer rated both twins. The internal consistency reliability of the interviewers' reports was 0.75. Data were available for 96% (*N* = 2,134) of the total sample.

Our rationale for including interviewers as informants was to gain information about children's antisocial behaviors in an unfamiliar, structured situation, with a stranger, where it would be expected to curb the inclination to behave antisocially. We thought of this setting as being representative of situations where the child needs to focus and behave appropriately. In addition, the interviewers assessed many children and were therefore able to evaluate children's behaviors relative to other children, providing an objective assessment.

#### Twins' Self‐Reports

We used the Berkeley Puppet Interview (BPI) to obtain self‐reports from the twins about their antisocial behaviors at age 5 (Arseneault, Kim‐Cohen, Taylor, Caspi, & Moffitt, 2005; Measelle, Ablow, Cowan, & Cowan, 1998). The BPI was administered to each twin separately. Children were asked 19 items covering three BPI scales that assess antisocial behaviors: overt aggression/hostility (e.g., “I fight with other kids”), conduct problems (e.g., “I take things that don't belong to me”), and oppositionality (e.g., “I don't do what my teacher asks me to do”). All interviews were videotaped to score the twins' answers later. Each item was coded on a 7‐point Likert scale ranging from 1 (*no symptom*) to 7 (*definite symptom*). Two different coders scored each interview, with interrater reliability exceeding 0.90 for all coders. The internal consistency reliability was 0.82. Data were available for 84% of the total sample (*N* = 1,879). We used a computerized questionnaire to obtain self‐reports of antisocial behaviors when the twins were 12 years old. All items were specifically selected to map onto the *DSM–IV* criteria for conduct disorder (APA, 1994). Items included the use of weapons (e.g., “Have you used a weapon on someone like a knife, piece of wood or baseball bat?”), truancy (e.g., “Do you sometimes skip school when you shouldn't?”), and stealing (e.g., “Have you stolen something while nobody was looking?”). Children responded with “yes” or “no” and were able to refuse to answer. The internal consistency reliability was 0.82. Data were available for 95% (*N* = 2,120) of the total sample.

There was no selective dropout at age 12 with regard to antisocial behaviors at age 5, irrespective of informant. Means, standard deviations, and ranges of mothers', teachers', interviewers', and twins' reports are reported in Table 1. We observed significant sex differences in levels of antisocial behaviors: Across all four measures, boys displayed higher levels of antisocial behaviors than girls. Information on age 5 measures has been published previously (Arseneault et al., 2003) and is reported in Table S1 in the online Supporting Information.

**Table 1:** Boys' and Girls' Antisocial Behaviors Rated by Mothers, Teachers, Interviewers, and Twins at Age 12

	Informants on twins' antisocial behaviors											
	Mothers			Teachers			Interviewers			Twins		
	*M* (*SD*)	Range	*N*	*M* (*SD*)	Range	*N*	*M* (*SD*)	Range	*N*	*M* (*SD*)	Range	*N*
Total	13.11 (11.60)	0–74	2,141	6.25 (11.18)	0–72	1,767	1.06 (1.70)	0–10	2,134	2.46 (2.94)	0–24	2,120
Boys	15.03 (12.61)	0–74	1,043	8.57 (12.72)	0–72	872	1.25 (1.85)	0–10	1,036	3.11 (3.38)	0–24	1,028
Girls	11.29 (10.23)	0–72	1,098	4.00 (8.89)	0–68	895	0.88 (1.52)	0–10	1,098	1.85 (2.29)	0–16	1,092

Note

The range reflects the observed range of values. Values are unstandardized; therefore, the means from different informants are not comparable. Gender differences were significant for each informant's report (all *p*s < .001).

### Genetic Models

We used the classic twin design (Neale & Cardon, 1992) to test the relative influence of genes and the environment on pervasive and situational antisocial behaviors at age 12 and its continuity since age 5. MZ twins are genetically identical, whereas DZ twins share, on average, 50% of their genes. Comparing the correlation of a phenotype within pairs of MZ and DZ twins allows to estimate the relative influence of additive genetic (A), shared environmental (C), and nonshared environmental (E) factors on behaviors. C represents environmental factors that make members of a family similar, while E represents factors that make members of a family different.

**Figure 1:**
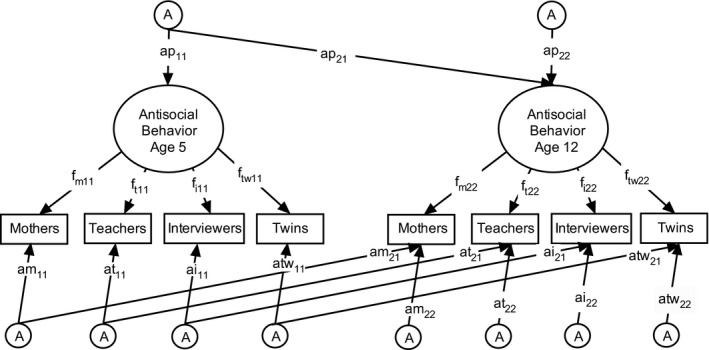
Longitudinal psychometric model for antisocial behaviors according to mothers', teachers', interviewers', and twins' reports. For illustrative purposes, this model only shows genetic influences (A). The actual model also included shared (C) and nonshared environmental influences (E). The latent factors of pervasive antisocial behaviors at Time 1 (age 5) and Time 2 (age 12) are influenced by genetic influences through paths ap_11_ and ap_22_. Paths f_m_, f_t_, f_i_, and f_tw_ are the factor loadings of each informant's report on the latent factor. The lower part of the model contains the variance in informants' reports that is not captured by the latent factor, and that is influenced by A through paths am, at, ai, and atw at both time points. The longitudinal paths (subscript 21) reflect genetic influences on antisocial behaviors at age 12 that are already influencing antisocial behaviors at age 5. The age 12 paths (subscript 22) reflect influences over and above those from age 5.

We used a multiple‐rater psychometric model with a longitudinal Cholesky decomposition to incorporate the information from all four informants at both time points (Figure 1). The psychometric model posits that genetic and environmental factors influence each of the four measures of antisocial behaviors via a latent factor. This factor captures agreement across informants, and therefore represents antisocial behaviors that are pervasive across settings. In contrast, informant‐specific variance reflects observations of twins' behaviors that were not agreed upon by all informants and therefore represent situational antisocial behaviors. Unsystematic measurement error is contained in the estimates of informant‐specific nonshared environmental influences, because it decreases similarity within pairs of MZ twins. Systematic error, such as informant bias in mothers' or interviewers' ratings, is contained in estimates of shared environmental influences on the informant‐specific variance, as it would increase similarity within pairs of twins, both for MZ and DZ twins. Therefore, significant genetic effects on the informant‐specific variance indicate that these unique observations of the twins' behaviors reflect variance associated with heritable behaviors as opposed to systematic or unsystematic error.

We chose the psychometric model because it enabled us to disentangle pervasive from situational antisocial behaviors. In addition, previous studies have consistently shown that it is the most suitable model when examining different informants' evaluations of twins' behaviors (Arseneault et al., 2003; Bartels et al., 2003; Hudziak et al., 2003). In addition, we used univariate genetic models to analyze genetic and environmental influences on antisocial behaviors separately for each informant.

To examine how much of the genetic and environmental influences on pervasive and situational antisocial behaviors at age 12 were already in place at age 5, we specified a Cholesky decomposition for all common and rater‐specific genetic and environmental components, which provides the estimates for the longitudinal paths in the psychometric model (Figure 1; all pathways with a 21 subscript, e.g., ap_21_ for genetic influences on pervasive antisocial behaviors, and am_21_, at_21_, ai_21_, and atw_21_ for informant‐specific observations). These paths indicate how much of the etiological influences on antisocial behaviors at age 12 were already present at age 5.

We assessed the stability of pervasive antisocial behaviors by examining their correlation between ages 5 and 12. To express the relative importance of genetic and environmental influences for the stability and the extent to which etiological influences at ages 5 and 12 correlate with each other, we transformed the Cholesky decomposition in Figure 1 into the mathematically identical correlated factors solution (Loehlin, 1996).

To test for sex differences, we first constrained all means to be equal for boys and girls. This led to a significant deterioration of fit (∆−2LL = 144.18, ∆*df *= 8, *p* < .001), indicating mean differences in antisocial behaviors between boys and girls. To test whether the factor loadings and the genetic and environmental influences varied according to sex, we constrained these paths to be equal for boys and girls and compared the fit to a model where only the means were allowed to vary across sex. We observed a significant deterioration of fit (∆−2LL = 161.47, ∆*df* = 51, *p* < .001), indicating overall differences in path estimates. We therefore present all path estimates for boys and girls separately. The differences in path estimates between boys and girls were not due to variance differences across sex, because including variance differences in the model did not affect the results or improve the fit of the model.

All variables were log‐transformed to normalize their distributions. All genetic analyses were conducted using the structural equation modeling program OpenMx (Boker, Neale, Maes, Wilde, & Spiegel, 2011). Missing data in the genetic analyses were handled using full information maximum likelihood (Baraldi & Enders, 2010). Information on genetic and environmental influences on age 5 measures has been published previously (Arseneault et al., 2003) and is reported in Table S2 in the online Supporting Information.

## Results

### Informants' Agreement on Twins' Antisocial Behaviors

Correlations between mothers', teachers', interviewers', and twins' ratings of antisocial behaviors at age 12 ranged from 0.20 (for interviewers and teachers) to 0.42 (for mothers and teachers; Table 2). Correlations between teachers' and mothers' reports of antisocial behaviors, and also between twins' and mothers' reports, were higher for boys than for girls.

**Table 2:** Internal Consistency Reliabilities and Correlations Between Mothers', Teachers', Interviewers', and Twins' Reports on Antisocial Behaviors at Age 12

	Boys				Girls			
	Mothers	Teachers	Interviewers	Twins	Mothers	Teachers	Interviewers	Twins
Mothers	0.94				0.93			
Teachers	0.42	0.97			0.28	0.95		
Interviewers	0.28	0.20	0.79		0.27	0.21	0.77	
Twins	0.36	0.41	0.23	0.86	0.30	0.31	0.20	0.77

Note

All correlations were significant, *p* < .001. There were significant sex differences in the correlation between teachers' and mothers' reports (*p *<* *.001) and twins' and teachers' reports (*p *=* *.01). Internal consistency reliability coefficients (Cronbach's alpha) are reported on the diagonal.

Ratings from all informants loaded onto one factor representing agreement between informants (Table 3, second and fifth columns). Factor loadings ranged from 0.36 for interviewers' reports of girls' antisocial behaviors to 0.66 for teachers' reports of boys' behaviors, with overall higher factor loadings for boys than for girls. By squaring the factor loadings it is possible to obtain the amount of variance in informants' reports that reflects their agreement on antisocial behaviors and that is indicative of behaviors that are pervasive across situations. The factor loadings indicated that this proportion ranged from 13% (for interviewers' reports of girls' behaviors) to 44% (for teachers' reports of boys' behaviors). The remaining variance was accounted for by informants' observations that were not agreed upon by all informants, and measurement error.

**Table 3:** Genetic and Environmental Contributions to Antisocial Behaviors at Age 12 According to Mothers', Teachers', Interviewers', and Twins' Reports

	Boys				Girls			
	Factor loading	A (95% CI)	C (95% CI)	E (95% CI)	Factor loading	A (95% CI)	C (95% CI)	E (95% CI)
Pervasive	—	0.70 (0.38–0.89)	0.12 (0.00–0.42)	0.18 (0.11–0.27)	—	0.24 (0.01–0.58)	0.68 (0.36, 0.91)	0.08 (0.01, 0.17)
Situational								
Mothers	0.58 (0.51–0.65)	0.36 (0.10–0.62)	0.31 (0.07–0.53)	0.33 (0.27–0.42)	0.52 (0.43–0.60)	0.66 (0.45–0.80)	0.09 (0.00, 0.30)	0.24 (0.19, 0.30)
Teachers	0.66 (0.59–0.73)	0.20 (0.03–0.51)	0.22 (0.00–0.41)	0.58 (0.46–0.72)	0.54 (0.45–0.63)	0.29 (0.00–0.60)	0.24 (0.00, 0.51)	0.46 (0.37, 0.58)
Interviewers	0.38 (0.31–0.46)	0.68 (0.44–0.77)	0.05 (0.00–0.26)	0.28 (0.23–0.34)	0.36 (0.27–0.45)	0.56 (0.34–0.72)	0.12 (0.00, 0.33)	0.32 (0.26, 0.38)
Twins	0.57 (0.50–0.63)	0.03 (0.00–0.21)	0.36 (0.20–0.46)	0.61 (0.52–0.70)	0.44 (0.35–0.52)	0.24 (0.01–0.37)	0.03 (0.00, 0.23)	0.73 (0.63, 0.85)

Note

The A, C, and E estimates for pervasive antisocial behaviors are interpretable as proportions of the latent variance. The A, C, and E estimates for situational antisocial behaviors are expressed as proportions of the unique measured variance. Estimates of situational antisocial behaviors are net of variance in pervasive antisocial behaviors. The estimates reflect the total genetic and environmental influences on pervasive and situational antisocial behaviors at age 12; that is, they are the sum of the 22 and 21 paths in Figure 1. The full model results, including the information from age 5, are reported in Table S2 in the online Supporting Information. A = genetic influences; C = shared environmental influences; E = nonshared environmental influences. CI = confidence interval.

### Genetic and Environmental Influences on Pervasive Antisocial Behaviors at Age 12

Findings revealed significant differences between boys and girls in the etiology of pervasive antisocial behaviors at age 12 (Table 3). For boys, the majority of variance in pervasive antisocial behaviors was accounted for by genetic influences (0.70; interpretable as 70% of variance). Shared environmental effects made a smaller contribution (0.12), as did nonshared environmental influences (0.18). For girls, a different pattern emerged: Shared environmental influences were the main factor explaining variability in girls' pervasive antisocial behaviors (0.68). Genes had a weaker influence (0.24), and nonshared environmental influences were also relatively small (0.08). This indicates a small but significant role of nonshared environmental experiences free of measurement error in the etiology of preadolescent pervasive antisocial behaviors for both boys and girls.

When examining each informant's report of antisocial behaviors at age 12 separately, overall approximately half of variance was accounted for by genetic influences (Table S3 in the online Supporting Information). Shared and nonshared environmental influences accounted for approximately one fifth and one third of variance. Differences between boys and girls were overall less pronounced, and minimal for mothers' reports.

### Genetic and Environmental Influences on Situational Antisocial Behaviors at Age 12

Both boys' and girls' situational antisocial behaviors were influenced by genetic factors, but the magnitude differed across informants and gender (Table 3). While genetic influences explained a relatively small proportion of variance in teachers' (0.20 for boys and 0.29 for girls) and twins' (0.03 for boys and 0.24 for girls) situation‐specific observations, they explained larger proportions in mothers' (0.36 for boys and 0.66 for girls) and interviewers' (0.68 for boys and 0.56 for girls) observations. These estimates of genetic influences are free of systematic and unsystematic measurement error, indicating that observations of twins' antisocial behaviors that are not agreed upon by all informants reflect reliable individual differences between participants in our study. The magnitude of nonshared environmental influences, which include unsystematic measurement error, was highest for twins' self‐reports (0.61 for boys and 0.73 for girls) while accounting for approximately one third of variance in the other reports. Shared environmental influences, including systematic measurement error, were very small overall, accounting for no more than one fifth of variance in informants' unique observations.

### Developmental Origins of Genetic and Environmental Influences

Results from the Cholesky decomposition indicated that a large proportion of the genetic and environmental influences that we observed on pervasive antisocial behaviors at age 12 were already in place by age 5 (Table 4). For both boys and girls, all of the shared environmental influences had their origins in early childhood. This indicates that shared environmental influences on antisocial behaviors are relatively stable from early childhood to preadolescence. The same was true for the majority of genetic influences, although some genetic influences only became evident at age 12. This was the case particularly for girls' pervasive antisocial behaviors, where half of the genetic influences had their origins in early childhood, and the other half was specific to age 12. Nonshared environmental influences on both boys' and girls' behaviors were mostly new at age 12.

**Table 4:** Results of the Longitudinal Cholesky Decomposition, Expressed as Proportions^a^ of Genetic and Environmental Influences on Antisocial Behaviors at Age 12 That Were Already in Place at Age 5

	Boys (%)			Girls (%)		
	A	C	E	A	C	E
Pervasive	73	100	11	46	100	25
Situational						
Mothers	11	71	09	36	100	04
Teachers	60	100	00	00	13	00
Interviewers	01	20	00	04	33	00
Twins	100	100	00	26	100	01

Note

Estimates of situational antisocial behaviors are net of variance in pervasive antisocial behaviors. A = genetic influences; C = shared environmental influences; E = nonshared environmental influences.

^a^The percentages indicate how much of a genetic or environmental influence on antisocial behaviors at age 12 originates at age 5. The higher the percentage, the more the influence was already in place at age 5, with the remainder specific to age 12. For example, for boys' pervasive antisocial behaviors, 73% of the genetic influences (A) at age 12 were already present at age 5. The remaining genetic influences (i.e., 27%) are specific to age 12. The full model results, including the untransformed Cholesky path estimates, are reported in Table S2 in the online Supporting Information.

Variance in situational antisocial behaviors was mostly accounted for by genetic and environmental influences that were newly emerging at age 12, rather than by influences that were already detectable at age 5 (Table 4). More specifically, although the proportion of variance that was explained by genetic influences already active at age 5 varied across informants, it was overall smaller than for pervasive antisocial behaviors. The small shared environmental influences on situation‐specific antisocial behaviors had their origins mostly in early childhood, whereas nonshared environmental influences on unique observations of antisocial behaviors were all specific to age 12.

### Stability of Pervasive Antisocial Behaviors Between Childhood and Preadolescence

The stability of pervasive antisocial behaviors between ages 5 and 12 was high both for boys (*r* = .74) and girls (*r* = .70; Table 5). For boys, the stability was mainly explained by genetic influences (0.88), with little contribution from nonshared environmental (0.08) or shared environmental (0.04) influences. The stability of girls' antisocial behaviors was mainly explained by shared environmental influences (0.55) and to a lesser extent by genetic influences (0.39). Nonshared environmental influences explained only a small proportion (0.06).

**Table 5:** Stability, and Genetic and Environmental Influences on Stability, of Pervasive Antisocial Behaviors Between Ages 5 and 12

	Boys	Girls
Stability between ages 5 and 12 (95% CI)	0.74 (0.65, 0.82)	0.70 (0.59, 0.80)
Proportion of stability accounted for by		
A (95% CI)	0.88 (0.54, 1)	0.39 (0.06, 0.73)
C (95% CI)	0.04 (0.00, 0.36)	0.55 (0.23, 0.85)
E (95% CI)	0.08 (0.00, 0.16)	0.06 (0.00, 0.15)
Genetic and environmental correlations^a^		
rA (95% CI)	0.85 (0.67, 1)	0.69 (0.18, 1)
rC (95% CI)	1 (0.00, 1)	1 (0.76, 1)
rE (95% CI)	0.35 (0.02, 0.67)	0.44 (0.00, 1)

Note

The stability is expressed as the correlation between the two latent factors at ages 5 and 12. A = genetic influences; C = shared environmental influences; E = nonshared environmental influences.

^a^These correlations indicate the degree to which the same genetic and environmental influences affect pervasive antisocial behaviors at ages 5 and 12. The higher the correlation, the more there is overlap between these influences across time.

## Discussion

We used a multi‐informant longitudinal twin design to extend previous findings on the genetic and environmental contributions to antisocial behaviors by examining pervasive and situational behaviors in preadolescence. Pervasive antisocial behaviors were mostly accounted for by genetic influences in boys and by shared environment in girls. For both genders, the majority of these influences had their origins in early childhood. Situational antisocial behaviors were not only more strongly influenced by nonshared environment in boys and girls, but also showed some genetic influences. In contrast to pervasive antisocial behaviors, the genetic and environmental influences on situational antisocial behaviors were mostly newly emerging in preadolescence.

### Pervasive Antisocial Behaviors Are Not Immune to Environmental Influences

Although early‐onset and persistent antisocial behaviors are known to be highly genetically influenced, our findings show that environmental influences are also important. We found substantial shared environmental influences on girls' antisocial behaviors, and to a smaller extent, on boys' also. Furthermore, our results show significant influences of nonshared environment for both boys and girls. This finding indicates that there are environmental influences even on pervasive antisocial behaviors. Identifying which factors these influences represent is a critical step toward improved interventions to reduce the social and economic burden of antisocial behaviors. Several studies have used genetically sensitive designs to identify environmental influences on antisocial behaviors, including negative parental discipline (Viding, Fontaine, Oliver, & Plomin, 2009), physical maltreatment (Jaffee, Caspi, Moffitt, & Taylor, 2004), and socioeconomic status (Odgers et al., 2012). It remains to be tested whether these can account for any variation in pervasive antisocial behaviors more specifically.

A previous study of the etiology of pervasive antisocial behaviors in our sample at age 5 did not show a significant contribution of shared environmental factors, indicating that these become more influential in preadolescence compared to childhood (Arseneault et al., 2003) and also adulthood (Rhee & Waldman, 2002). This finding is consistent with other research and suggests a temporary increase in the importance of shared environmental influences on antisocial behaviors during preadolescence (Silberg, Rutter, Tracy, Maes, & Eaves, 2007).

### Sex Differences

Although the magnitude of familial influences on pervasive antisocial behaviors was similar across gender, boys' behaviors were mostly accounted for by genetic influences, whereas girls' behaviors were better explained by shared environmental influences. Other studies have obtained similar findings, particularly when examining pervasive and persistent antisocial behaviors (Bartels et al., 2011; Fontaine et al., 2010; Hudziak et al., 2003). This suggests that sex differences are most pronounced when pervasive and persistent antisocial behaviors are examined, and could explain why some other studies, including one meta‐analysis, did not find sex differences (Rhee & Waldman, 2002). It would also be consistent with the less pronounced differences between boys and girls in the univariate estimates in our study, with virtually no sex differences in mothers' reports.

There are other explanations for the sex differences we found. First, it is possible that differences in boys' and girls' pubertal development account for some of our findings. For example, girls enter puberty earlier than boys (Tanner, 1981), and puberty has been associated with a temporary increase in shared environmental influences on antisocial behaviors in some studies (Silberg et al., 2007). Thus, it is possible that the stronger role of shared environmental influences in girls during preadolescence reflects to some extent their advanced pubertal development. Second, it has been found that girls are monitored by their parents more than boys in adolescence (Racz & McMahon, 2011). Parental monitoring and other differential parenting may present shared environmental influences that affect girls' antisocial behaviors more than boys'. Less monitoring and less parental restriction of boys' behaviors may also translate into less environmental constraint on the expression of their genetic propensities, so that boys' genetic influences have more freedom to manifest. In addition, less monitoring gives boys more opportunities to select environments consistent with their genetic dispositions. In a twin design, such gene–environment interplay would lead to increased estimates of genetic influences. Third, it has been suggested that boys are more vulnerable to shared environmental risk factors such as economic stress and family discord (Rutter, Giller, & Hagell, 1998). If such vulnerability is in part genetically mediated, it may result in gene‐environment interactions, which would be reflected in estimates of genetic influences. Therefore, larger environmental influences on girls' antisocial behaviors may not all be due to a stronger role of environment, but could also indicate sex differences in the genetic vulnerability to environmental influences, as has been demonstrated for genetic variants (Byrd & Manuck, 2014).

There were also some sex differences in the etiology of situational antisocial behaviors, with girls' behaviors more strongly influenced by genetic factors than boys'. The higher genetic influence we find on girls' situational antisocial behaviors could be explained by the lower agreement between informants for girls' behaviors, reflected in lower correlations across reports and lower factor loadings for girls in our study. Lower agreement may indicate that girls display antisocial behaviors less consistently across situations (Gray et al., 2012). Therefore, little of their behavior will be captured by informants' agreement (i.e., the latent factor), leaving informants' unique to contain more information about girls' individual differences, reflected in higher genetic influences. In contrast, the agreement between informants was higher for boys' antisocial behaviors. Their individual differences are therefore captured to a greater extent in the informants' agreement than in informants' unique observations, leaving these ratings to contain less meaningful variation and more error. These findings suggest that it might be easier to gain a more accurate picture of boys' pervasive antisocial behaviors even if information is only available from a few informants, whereas a complete evaluation of girls' antisocial behaviors may require information from several sources.

### The Majority of Influences on Pervasive Antisocial Behaviors Originate Earlier in Life

We showed that individual differences in pervasive antisocial behaviors in preadolescence were mostly accounted for by genetic and shared environmental influences that were already in place at age 5. Our finding indicates a high stability of genetic and shared environmental influences across time, which can explain the continuity of antisocial behaviors over the years. This is consistent with other studies examining the continuity of antisocial behaviors (Van Beijsterveldt, Bartels, Hudziak, & Boomsma, 2003), but extends previous research by studying pervasive antisocial behaviors. Stable genetic influences may reflect early‐life risk factors for later antisocial behaviors, such as a difficult temperament, hyperactivity and impulsivity, and deficits in neurocognitive functioning (Caspi et al., 1995; Farrington, 2005).

Stable shared environmental influences may reflect aspects of parenting (Burt, 2009; Dallaire & Weinraub, 2005). Interestingly, shared environmental influences were small and unimportant for both boys and girls in our sample at age 5 (Arseneault et al., 2003), and accounted for a substantial proportion of variability in girls' antisocial behaviors only at age 12. This suggests that even if shared environmental influences on antisocial behaviors are small in early childhood, they cannot be disregarded as they might affect antisocial behaviors later in life (Burt, 2009).

### Dynamic Genome and Dynamic Environment

While a substantial proportion of genetic influences on pervasive antisocial behaviors at age 12 originated in early childhood, we also found evidence for a “dynamic genome” with new genetic influences emerging over time (Silberg et al., 2007; Van Hulle et al., 2009). Newly emerging genetic influences may be due to genes that are relevant to antisocial behaviors being expressed over time, for example, in response to environmental changes or biological maturation during preadolescence (Van Hulle et al., 2009). Because estimates of genetic influences obtained from a twin design contain some forms of gene–environment interplay, it is also possible that the newly emerging genetic influences capture some of this interplay. For example, it is possible that children's genetic vulnerability for antisocial behaviors makes them more likely to affiliate with deviant peers during preadolescence (Moffitt, 2005). Our findings also provide evidence for a “dynamic environment,” whereby novel nonshared environmental influences appear over time. Although the time specificity of nonshared environmental influences has been found in other research as well (Van Beijsterveldt et al., 2003; Van Hulle et al., 2009), our study shows that it remains even when nonshared environmental influences are unconfounded by unsystematic measurement error. These influences may reflect new child‐specific experiences that account for variance in antisocial behaviors, for example, exposure to violence during preadolescence.

It is important to note that estimates of genetic and environmental influences obtained from twin studies may include processes of gene–environment interplay. Interactions between genetic effects and shared environments increase estimates of genetic influences, whereas interactions with nonshared environments elevate estimates of nonshared environment. Active or evocative gene–environment correlations, whereby children select or evoke environments based on their genetic propensities, increase genetic influences. Passive gene–environment correlations, whereby parents' genes influence the environment they provide for their children and their children's behaviors, increase estimates of shared environment. All of these forms of gene–environment interplay are involved in the etiology of antisocial behaviors (Moffitt, 2005). It is therefore important to interpret etiological influences not as exclusively genetic or environmental, but to be mindful of considering processes of gene–environment interplay in research and clinical practice.

### Limitations

Our findings must be interpreted in light of some limitations. First, the interviewer measure at age 12 assessed aggressive behaviors and not general antisocial behaviors. The factor loadings show that the interviewer measure did not reflect pervasive antisocial behaviors to the same extent as the other informants' reports. However, factor loadings were still moderate, reflecting a degree of agreement with other informants that is remarkable considering that interviewers interacted with twins for a few hours. Second, antisocial behavior is a broad construct and it has been suggested that subtypes of antisocial behaviors may differ in their genetic and environmental etiology (Caspi et al., 1995; Eley, Lichtenstein, & Moffitt, 2003). However, a meta‐analysis did not find any differences, whether antisocial behaviors were defined as conduct disorder, criminality and delinquency, or aggressive behaviors (Rhee & Waldman, 2002). Third, our sample comprised twins and we cannot be certain that our results generalize to singletons. However, twins versus singletons are not different in their prevalence rates of antisocial behaviors or antisocial personality traits (Johnson, Krueger, Bouchard, & McGue, 2002; Moilanen et al., 1999) and the effect sizes for associations between risk factors and psychopathology outcomes have generally been found to be similar across behavioral genetic and nongenetic studies (Moffitt & E‐Risk Study Team, 2002). Fourth, we cannot be certain that the latent factors in our study only reflect antisocial behaviors. They may include other behaviors or characteristics that informants agree on, such as impulsivity or negative emotionality. Finally, we did not explicitly assess antisocial behaviors across different settings. Instead, we inferred pervasiveness and situation specificity from agreement and disagreement between informants who observe or interact with children in different contexts. Although this strategy has ample empirical support, a more direct approach would be for one external observer to record children's antisocial behaviors across different settings (e.g., Wakschlag et al., 2008). Once such a measure is used in a genetically sensitive design, it will be interesting to compare the findings across these different operationalizations of pervasiveness.

### Implications

Our findings have implications for the prevention and treatment of children's antisocial behaviors. First, our results show that pervasive antisocial behaviors are affected by familial influences—both genetic and environmental—suggesting that it is important to integrate the family environment of children into prevention and treatment efforts (Scott et al., 2009). It may be particularly promising to focus on antisocial parents, since they not only pass on a genetic risk to their children, but also provide a caregiving environment that could promote antisocial behaviors (Moffitt, 2005). Second, situational antisocial behaviors reflect meaningful individual differences in children's antisocial behaviors, and may also require treatment. Third, our finding that influences on pervasive antisocial behaviors originate mostly in early childhood emphasizes the importance of early intervention. In clinical or research settings, longitudinal measurements are often lacking and as a result it is difficult to establish whether a child's antisocial behavior pattern is early‐onset persistent. In such settings, cross‐sectional agreement across reporters from multiple settings can substitute, providing similar information about etiology, severity, and treatment need. To date, cross‐situational pervasive symptoms are required for diagnosis of attention deficit hyperactivity disorder, but not conduct disorder.

Our study also has implications for research on the etiology of antisocial behaviors. We showed that mothers, teachers, interviewers, and children all contribute meaningfully to the measurement of antisocial behaviors. Where possible, collecting information from all of these informants will help compile a comprehensive assessment of children's behaviors. However, the findings also show that even sole informants, particularly teachers and mothers, provide information on children's pervasive antisocial behaviors. Furthermore, the genetic influence on situation‐specific antisocial behaviors shows that informants' unique observations of antisocial behaviors may provide valuable information about a child's behaviors, and cannot be discarded as reflecting measurement error.

Our findings can help to guide further research on the etiology and outcomes of antisocial behaviors. We showed that there are environmental influences on pervasive antisocial behaviors. A next step would be to identify which specific environmental characteristics influence pervasive antisocial behaviors, and how these differ between boys and girls. In addition, it will be interesting to examine to what extent genetic influences on pervasive antisocial behaviors reflect gene–environment interplay (Moffitt, 2005). Finally, our findings highlight the relevance of situation‐specific antisocial behaviors. We need more research to examine the extent to which pervasive and situation‐specific antisocial behaviors differ in their outcomes, and the risk factors leading children to show behaviors in some settings, but not others.

## Supporting information


**Table S1.** Boys' and Girls' Antisocial Behaviors Rated by Mothers, Teachers, Interviewers, and Twins at Age 5.
**Table S2.** Results of Longitudinal Psychometric Model With Cholesky Decomposition
**Table S3.** Univariate Estimates of Genetic and Environmental Influences for Mothers', Teachers', Interviewers', and Twins' Reports of Antisocial Behaviors at Age 12
