# Supplementary material for: Etiology of Pervasive Versus Situational Antisocial Behaviors: A Multi‐Informant Longitudinal Cohort Study
Source: Child Dev. 2015 Nov 12;87(1):312–25. doi: 10.1111/cdev.12456 (PMC4949514; doi:10.1111/cdev.12456)
Supplement: Supplementary file 1 — Table S1. Boys' and Girls' Antisocial Behaviors Rated by Mothers, Teachers, Interviewers, and Twins at Age 5. Table S2. Results of Longitudinal Psychometric Model With Cholesky Decomposition Table S3. Univariate Estimates of Genetic and Environmental Influences for Mothers', Teachers', Interviewers', and Twins' Reports of Antisocial Behaviors at Age 12 [file CDEV-87-312-s001.docx]

**Supporting Information for Online Publication Only**

Table S1 *Boys’ and girls’* *antisocial behaviors rated by mothers, teachers, interviewers and twins at age 5.*

|  | Informants on twins’ antisocial behaviors at age 5 | | | | | | | | | | | | | | | | | | | | | |
| --- | --- | --- | --- | --- | --- | --- | --- | --- | --- | --- | --- | --- | --- | --- | --- | --- | --- | --- | --- | --- | --- | --- |
|  | Mothers | | | |  | | Teachers | | | |  | | Interviewers | | | |  | | Twins | | | |
|  | M (SD) | Range | N |  | | M (SD) | | Range | N |  | | M (SD) | | Range | N |  | | M (SD) | | Range | N |  |
| Total | 16.87 (12.13) | 0-72 | 2230 |  | | 6.18 (9.63) | | 0-74 | 2091 |  | | 2.41 (3.63) | | 0-18 | 2225 |  | | 52.54 (14.09) | | 31-106 | 1879 |  |
| Boys | 18.72 (13.21) | 0-72 | 1092 |  | | 7.89 (11.12) | | 0-74 | 1025 |  | | 3.06 (4.00) | | 0-18 | 1086 |  | | 54.77 (14.92) | | 31-106 | 890 |  |
| Girls | 15.09 (10.71) | 0-68 | 1138 |  | | 4.52 (7.59) | | 0-66 | 1066 |  | | 1.78 (3.12) | | 0-18 | 1139 |  | | 50.53 (12.99) | | 31-103 | 989 |  |

*Note*: The range reflects the observed range of values. Values are unstandardized, therefore the means from different informants are not comparable. Gender differences were significant for each informant’s report (all p<.001).

Table S2 *Results of longitudinal psychometric model with Cholesky decomposition.*

|  |  | Boys | | | |  | Girls | | | |
| --- | --- | --- | --- | --- | --- | --- | --- | --- | --- | --- |
|  |  | Factor loading | A (95% CI) | C (95% CI) | E (95% CI) |  | Factor loading | A (95% CI) | C (95% CI) | E (95% CI) |
| **Age 5** | Pervasive | - | .83 (.51, .92) | .01 (.00, .31) | .16 (.08, .26) |  | - | .67 (.41, .87) | .22 (.05, .45) | .11 (.03, .22) |
|  | Situational |  |  |  |  |  |  |  |  |  |
|  | Mothers | .57 (.49, .64) | .32 (.06, .60) | .32 (.06, .55) | .36 (.29, .44) |  | .44 (.35. .52) | .61 (.41, .71) | .04 (.00, .22) | .35 (.29, .42) |
|  | Teachers | .50 (.41, .58) | .72 (.56, .79) | .03 (.00, .17) | .25 (.20, .31) |  | .55 (.45, .65) | .57 (.30, .75) | .11 (.00, .36) | .31 (.25, .40) |
|  | Interviewers | .37 (.28, .45) | .41 (.17, .65) | .21 (.00, .42) | .38 (.32, .46) |  | .35 (.26, .44) | .47 (.23, .66) | .14 (.00, .36) | .39 (.33, .46) |
|  | Twins | .34 (.25, .43) | .22 (.00, .37) | .10 (.00, .31) | .68 (.58, .80) |  | .42 (.33, .52) | .23 (.00, .44) | .11 (.00, .33) | .66 (.55, .79) |
| **Cholesky paths** | Pervasive |  | .51 (.24, .84) | .12 (.00, .42) | .02 (.00, .08) |  |  | .11 (.00, .33) | .68 (.32, .91) | .02 (.00, .11) |
|  | Situational |  |  |  |  |  |  |  |  |  |
|  | Mothers | - | .04 (.00, .26) | .22 (.02, .50) | .03 (.01, .06) |  | - | .24 (.10, .41) | .09 (.00, .30) | .01 (.00, .02) |
|  | Teachers | - | .12 (.02, .29) | .22 (.00, .41) | .00 (.00, .03) |  | - | .00 (.00, .12) | .03 (.00, .49) | .00 (.00, .01) |
|  | Interviewers | - | .01 (.00, .16) | .01 (.00, .20) | .00 (.00, .02) |  | - | .02 (.00, .14) | .04 (.00, .30) | .00 (.00, .01) |
|  | Twins | - | .03 (.00, .20) | .36 (.02, .46) | .00 (.00, .01) |  | - | .06 (.00, .35) | .03 (.00, .23) | .01 (.00, .03) |
| **Age 12** | Pervasive |  | .19 (.00, .41) | .00 (.00, .32) | .16 (.08, .24) |  |  | .13 (.00, .38) | .00 (.00, .28) | .07 (.00, .15) |
|  | Situational |  |  |  |  |  |  |  |  |  |
|  | Mothers | .58 (.51, .65) | .32 (.10, .51) | .09 (.00, .32) | .31 (.24, .38) |  | .52 (.43, .60) | .42 (.25, .53) | .00 (.00, .16) | .23 (.19, .29) |
|  | Teachers | .66 (.59, .73) | .08 (.00, .45) | .00 (.00, .41) | .58 (.45, .71) |  | .54 (.45, .63) | .29 (.00, .59) | .21 (.00, .49) | .46 (.37, .58) |
|  | Interviewers | .38 (.31, .46) | .67 (.42, .75) | .03 (.00, .25) | .28 (.23, .33) |  | .36 (.27, .45) | .54 (.32, 72) | .08 (.00, .32) | .32 (.26, .38) |
|  | Twins | .57 (.50, .63) | .00 (.00, .19) | .00 (.00, .38) | .61 (.52, .70) |  | .44 (.35, .52) | .17 (.00, .36) | .00 (.00, .23) | .73 (.62, .85) |

*Note:* A=genetic influences, C= shared environmental influences, E= non-shared environmental influences. Cholesky path estimates indicate how much of the A, C, E influences on age-5 antisocial behavior also explain variability in age-12 behavior. Age-12 estimates indicate the A, C, E influences specific to age 12, independent of age-5 influences. The total A, C, E influences on age-12 behavior are the sum of the Cholesky path and age-12 estimates. All estimates are interpretable as proportions of variance.

Table S3 *Univariate estimates of genetic and environmental influences for mothers’, teachers’, interviewers’ and twins’ reports of antisocial behaviors at age 12.*

|  | Total | | | | |
| --- | --- | --- | --- | --- | --- |
|  | A (95% CI) | | C (95% CI) | | E (95% CI) |
| Mothers | .55 (.42-.69) | | .22 (.09-.34) | | .23 (.20-.26) |
| Teachers | .38 (.20-.56) | | .27 (.10-.42) | | .35 (.31-.41) |
| Interviewers | .57 (.43-.73) | | .16 (.01-.29) | | .27 (.24-.30) |
| Twins | .17 (.00-.36) | | .30 (.14-.46) | | .52 (.47-.59) |
|  | Boys | | | | |
|  | A (95% CI) | C (95% CI) | | E (95% CI) | |
| Mothers | .52 (.33-.73) | .21 (.00-.38) | | .28 (.23-.33) | |
| Teachers | .47 (.20-.67) | .13 (.00-.37) | | .39 (.32-.48) | |
| Interviewers | .65 (.45-.78) | .09 (.00-.28) | | .26 (.21-.31) | |
| Twins | .06 (.00-.32) | .42 (.20-.53) | | .52 (.44-.60) | |
|  | Girls | | | | |
|  | A (95% CI) | C (95% CI) | | E (95% CI) | |
| Mothers | .59 (.43-.78) | .21 (.02-.37) | | .20 (.17-.24) | |
| Teachers | .28 (.05-.53) | .36 (.13-.56) | | .36 (.30-.44) | |
| Interviewers | .48 (.29-.70) | .23 (.03-.41) | | .29 (.24-.34) | |
| Twins | .30 (.02-.49) | .11 (.00-.35) | | .59 (.50-.68) | |

*Note:* A = genetic influences, C = shared environmental influences, E= non-shared environmental influences. For each informant, a model assuming sex differences fit better than a model constraining boys’ and girls’ estimates to be equal. All estimates are interpretable as proportions of variance.
